# GRAPHOS re-examined: graph-theoretic features do not improve dyslexia screening, and a demographic confound in the Dytective dataset

**DOI:** 10.3389/fdgth.2026.1927725

**Published:** 2026-09-23

**Authors:** Sudha M, Murali S

**Affiliations:** 1 School of Advanced Sciences (SAS), Vellore Institute of Technology, Vellore, Tamil Nadu, India; 2 School of Computer Science and Engineering (SCOPE), Vellore Institute of Technology, Vellore, Tamil Nadu, India

**Keywords:** dyslexia screening, evaluation methodology, graph-theoretic analysis, model selection, negative result, permutation control, sample-size confound, task correlation networks

## Abstract

**Introduction:**

Most machine learning methods for dyslexia screening treat the performance measurements of cognitive tasks as independent feature vectors. It has been hypothesised that this discards information carried by the interdependence among tasks probing shared cognitive processes. This paper tests that hypothesis and does not find support for it.

**Methods:**

We evaluate GRAPHOS, a graph-theoretic framework that builds task correlation networks from gamified cognitive assessment data, on the Dytective dataset of 3,644 participants (392 dyslexic). Model, aggregation strategy and decision threshold are selected exclusively on training and validation partitions, and the test partition is read once.

**Results:**

On the threshold-free AUC, GRAPHOS does not outperform a strong single-classifier baseline (0.8881 vs. 0.8883; DeLong p = 0.989), and this null replicates across ten random splits. A model using no graph-derived features is statistically indistinguishable from the full framework. A size-matched permutation null shows that none of nine population-level network differences between the dyslexic and non-dyslexic groups survives correction for the 8.3:1 sampling asymmetry. A subgroup audit shows that dyslexia prevalence in this dataset differs 4.7-fold by native-language status, that four demographic columns alone reach AUC 0.724 against 0.744 for all 192 task metrics, and that including them yields a twelve-fold disparity in false-positive rate between language groups.

**Discussion:**

A psychometric analysis indicates why the graph representation gains nothing: the battery exhibits little common variance in this sample (KMO 0.497, Cronbach's α 0.171), and exploratory factor analysis recovers 13% of variance with 28 of 32 tasks loading on no factor. We report these as negative results together with the two controls that produced them, and suggest both as routine: the size-matched null wherever correlation networks are compared between groups of unequal size, and the subgroup audit wherever a screening classifier is trained on a sample whose label prevalence may covary with demographic composition.

## 1 Introduction

Dyslexia is a neurodevelopmental disorder of neurobiological origin which presents as

(a) persistent difficulties in recognizing words both correctly and fluently, poor spelling, and
(b) deficient decoding skills, in spite of adequate intelligence, motivation, and educational opportunity (1).

Various epidemiological studies suggest that the disorder ranges from 5% to 17% of the population globally depending on the diagnostic criteria and orthographic transparency of the language under consideration (2). The negative outcomes of undetected dyslexia are not only limited to academic underachievement. Without early recognition of children’s reading difficulties during the initial school years, they usually develop secondary emotional and behavioral problems, such as anxiety, low self-esteem, and school avoidance, which intensify the original learning difficulty and cause a considerable amount of long-term socioeconomic costs (3). Early identification is therefore widely recognized as the most crucial factor in preventing the worsening of the condition, providing an opportunity for intervention at a time of highest neural plasticity.

Nowadays, dyslexia assessment is very costly in terms of materials and human resources, as trained professionals, a large number of one-on-one evaluation sessions, and standardized psychometric tools are involved. Besides, such tools may not be accessible in educational settings that are poorly equipped or in less widely spoken languages that have no validated adaptations. Launching digital technology and gamified assessment platforms continue to offer an encouraging route to broad automated, screening.

The Dytective platform, an online gamified test administered to over 3,600 participants, and recall for dyslexic class stayed above 80% with Random Forest classifiers applied to interaction metrics across 32 linguistic tasks, were a demonstration of the feasibility of such a method, as presented by Rello et al. (4). Through that work, a major benchmark was set and a publicly available dataset that has since been the basis for a myriad of follow-up studies has been provided. However, a deep-seated restriction of these approaches, as well as of most existing computational methods for dyslexia detection, is that they are reliant on flat feature representations that consider each task metric as an independent predictor, while neglecting the structured relationships among tasks that stem from them probing overlapping cognitive resources like phonological processing, rapid automatization, and working memory.

Over the past ten years, the use of graph-theoretic and network science techniques for biomedical classification has received a lot of attention. In neuroscience, the analysis of brain connectivity usually involves the mapping of cortical and subcortical areas as nodes while statistical dependencies are considered as edges, thus showing organizational principles like small-world topology, modular community structure, and hierarchical hub architecture which vary systematically between healthy and clinical populations (5–7). Graph neural networks have been used on neuroimaging data for the classification of brain disorders with promising results Alzheimer’s disease to schizophrenia (8). Persistent homology in topological data analysis has been able to uncover shape-based features in high-dimensional biomedical datasets which are not discernible by conventional statistical descriptors (9, 10). Graph signal processing is effectively the extension of classical spectral analysis to irregular domains which allows one to break down signals defined on networks into components that correspond to smooth vs. rapidly varying patterns (11, 12). However, none of the existing frameworks has methodically combined network motif discovery, topological persistence, graph spectral analysis, hierarchical decomposition, robustness characterization, and multi-resolution analysis in a single classification pipeline for any screening application, let alone dyslexia detection from cognitive assessment data.

We address that question by presenting GRAPHOS (Graph-theoretic Reasoning and Analysis for Predictive Health Outcome Screening), a framework that builds task correlation networks from gamified cognitive assessments and extracts graph-theoretic features from several analytical paradigms. The name reflects the framework’s original design intent; as the results below show, we do not find that it improves screening performance, and we retain the name for continuity with the earlier submission rather than as a description of what it achieves. Instead of considering each of the 32 assessment tasks as an independent measurement, GRAPHOS constructs the weighted network of Spearman correlations among tasks and then uses six different graph-analytic components to depict the structural organization of cognitive performance for dyslexic and non-dyslexic populations. The main argument behind this method is that dyslexia results in the disruption of not only individual cognitive abilities but also the integration of different cognitive processes—which is something that network-level analysis can capture but is ignored by feature-level approaches. Classification features at the individual level are generated by mapping each participant’s performance profile onto population-level network structures, and the final predictions are the output of a multi-strategy ensemble classifier equipped with McNemar-optimized threshold selection, a procedure that is consistent with the statistical testing guidelines of Dietterich (13).

Evaluated on the Dytective dataset of 3,644 participants under a protocol in which the test partition is read once, the GRAPHOS ensemble does not outperform a strong single-classifier comparator on the threshold-free AUC (0.8881 vs. 0.8883; p=0.989), and the null replicates across ten random splits. A size-matched permutation control shows further that the population-level network differences between dyslexic and non-dyslexic task networks—lower transitivity, lower modularity, higher global efficiency—are not identifiable once the 8.3:1 sampling asymmetry between the groups is accounted for. We report both as negative results, together with the controls that produced them.

### 1.1 Contributions

The major contributions of this work are summarized as follows:

- **Novel graph-theoretic framework for dyslexia detection**:This paper presents GRAPHOS as the first framework that completely changes the way dyslexia screening is thought of and treated as a graph-theoretic network analysis problem. GRAPHOS is composed of eight specialized components that extract network motif, topological, spectral, hierarchical, robustness, and multi-resolution features from task correlation networks constructed from gamified cognitive assessment data.
- **Integration of complementary graph-analytic paradigms:** The proposed approach brings together eight distinct graph-analytic paradigms in a single classification pipeline: network motif discovery (triadic and tetradic patterns with significance testing), persistent homology-inspired topological analysis (Betti numbers, filtration curves, cycle basis characterization), graph signal processing (graph Fourier transform, spectral energy distribution, signal smoothness measures), k-core and onion decomposition with rich club analysis, network robustness simulation (targeted attack and random failure tolerance), and graph wavelet analysis. We further report a structural audit of this pipeline: of the eight components, only Graph Signal Processing and Hierarchical Graph Decomposition yield individual-level features. Network Motif Discovery, Topological Feature Analysis, Network Robustness Analysis and Multi-Resolution Analysis are population-level descriptive analyses and contribute no columns to the classifier. Of 282 features, 19 (6.7%) are graph-derived; the remainder are 192 raw task metrics, 4 demographic variables and 67 conventional per-participant summary statistics.
- **A leakage-free evaluation protocol and a negative classification result:** We specify a protocol in which the comparator, the aggregation strategy and the decision threshold are all selected without reference to test labels, and in which the same threshold-selection rule is applied to every arm. Under this protocol GRAPHOS does not improve on a strong single-classifier baseline on the threshold-free AUC (p=0.989), and the null replicates across ten random splits. We further show that a threshold-dependent McNemar statistic computed on a single split is sign-unstable: of five splits reaching p<0.05, three favour GRAPHOS and two favour the baseline.
- **Identification of a demographic confound in a widely used dyslexia dataset:** We show that label prevalence in the Dytective sample differs 4.7-fold by native-language status, that four demographic columns alone reach 66.3% of the full model’s discrimination above chance, and that their inclusion produces a twelve-fold between-group disparity in false-positive rate. We supply the subgroup audit that establishes this and recommend it wherever a screening classifier is trained on a sample whose label prevalence may covary with demographic composition.
- **A size-matched null for group comparisons of correlation networks:** We show that comparing networks estimated from groups of unequal size at a fixed correlation threshold is confounded by construction, because noisier correlation estimates from the smaller group scatter edges rather than clustering them. Against a null built from 1,000 size-matched subsamples, none of nine population-level differences survives. We recommend this control wherever group correlation networks are compared.

The rest of this paper is structured as follows. Section 2 provides a thorough review of the related works as well as identification of research gaps. Section 3 introduces the GRAPHOS framework in depth through network construction and all eight analytical components with mathematical formulations. Section 4 details the experimental set-up. Section 5 shows the experimental results. Section 6 discusses the findings, clinical implications, and limitations. Section 7 wraps up the paper with conclusions and suggests directions for future research.

## 2 Related work

### 2.1 Machine learning for dyslexia detection

Computational methods for dyslexia screening with various data types have dramatically increased over the last decade.

Rello et al. (4) made a major contribution in this area by proving the feasibility of screening dyslexia through a gamified web test. They gathered Spanish-speaking participant data from 3,600+ individuals working on 32 language tasks. Six performance variables per task were recorded. Random Forest classifiers used by their work gave recall above 80% for the dyslexic category on the desktop data and 78% on a tablet data. This study also released the Dytective dataset which is used in our research.

Alkhurayyif and Sait (14) carried out an extensive review of AI methods for dyslexia detection. They included dimensionality reduction combined with machine learning and deep learning architectures in their survey. Their paper concluded that the leading paradigms are convolutional neural networks applied to handwriting images, recurrent networks for eye-tracking sequences, and ensemble classifiers for behavioral metrics. However, a fact discovered was that most methods handle individual task measures isolatedly and do not involve exploiting inter-task correlations.

Eroğlu (15) examined EEG-based machine learning methods for dyslexia biomarker detection and identified classification accuracy levels from 82% to 99.2% in 15 studies conducted between 2013 and 2025. However, he also pointed out that generalizability is a major issue due to small sample sizes and heterogeneous protocols across studies.

One of the biggest issues highlighted by the reviewed literature is that cognitive tasks’ relational structure is not modeled with methods. For instance, if a set of tasks indexes phonological awareness, rapid naming, working memory and visual processing, then the correlation structure of task performance might carry information about how those processes relate that flat feature vectors discard. We state this as the hypothesis motivating the present work rather than as an established premise, and we note in advance that our results do not support it: a between-participant correlation between two task scores is a statement about covariation in performance, not about interaction between cognitive subsystems, and Section 6.2 sets out why we no longer make the stronger reading. Flat feature representations completely discard this structural information which is the motivation behind the network based model implemented in GRAPHOS.

### 2.2 Graph-theoretic analysis in biomedical applications

Analyzing complex networks has become a basic method in neuroscience and medical research. Bullmore and Sporns (5) authored a landmark paper that comprehensively addressed the importance of graph-theoretical parameters such as clustering coefficient, average shortest path length, modularity, and the identification of hubs in brain networks for neurological disease understanding. Rubinov and Sporns (6) came up with a full-fledged set of network analysis metrics for brain connectivity along with clear guidelines for interpretation, which have become a standard in the field.

Watts and Strogatz (7) first demonstrated the small-world phenomenon. Brain networks exhibiting this property have regularly appeared in the literature. On the contrary, alterations of this typical pattern have been associated with several neurological and psychiatric disorders (8).

In a review, Mohammadi and Karwowski (8) focused on the use of graph neural networks for classification based on brain connectivity. They especially pointed out the progress made in integrating different modalities and modelling dynamic connectivity. Li et al. (16) presented a thorough review of graph signal processing and graph learning in the realm of biological data. They concluded that graph methods sufficiently capture the complex interrelations at various biological levels. Calazans et al. (17) went through machine learning and graph signal processing applications in healthcare, found 45 studies that were conducted since 2015, and described the healthcare domain as an emerging one that is rapidly expanding. Although these reviews provide the theoretical background on which GRAPHOS is based, none of them applies integrated multi-paradigm graph analysis specifically to cognitive assessment data.

### 2.3 Network motifs and topological data analysis

A motif in a network can be described as a pattern of subgraphs that occur repeatedly with significantly high frequency as compared to their expected appearance in random networks. Milo et al. (18) were the first to introduce the concept of network motifs as the basic elements of complex networks. Generally, different classes of networks are characterized by different types of motif patterns or profiles, which in turn, indicates that motif analysis can reveal the functional organization that even the aggregate statistics cannot. Kashtan et al. (19) came up with very efficient sampling algorithms making it possible to carry out motif significance testing on a large scale by comparing with degree-preserving random ensembles.

On the other hand, topological data analysis with a special focus on persistent homology (10, 20) offer a different perspective by describing the shape of data at different resolutions. For example, Singh et al. (9) gave an overview of how TDA has been used in medical imaging. They showed that the features based on persistence are able to capture the structural information which is not accessible by the conventional descriptors. De Lara (21) put forward a classification method based only on persistent homology which was able to achieve performance comparable to the standard classifiers. GRAPHOS reinterprets these ideas by correlation matrix filtration analysis that monitors the changes of topological features as the correlation threshold is varied.

### 2.4 Hierarchical decomposition, robustness, and graph signal processing

K-core decomposition helps in pinpointing the largest possible subgraphs of increasing minimum degree, thus uncovering a hierarchical core-periphery pattern (22). Hagmann et al. (23) utilized k-core analysis on the structural connectome and found that the cerebral cortex indeed has a defining structural core. Rich club organization—the propensity of high-degree nodes to connect with each other—has been identified as one of the main features of a healthy brain architecture (24) Network robustness analysis looks at the functional decline after a perturbation; Albert et al. (25) discovered that scale-free networks have the unique property of being robust against random failures while being vulnerable to targeted attacks on hubs simultaneously.

Latora and Marchiori (26) proposed global and local efficiency as measures of information transfer capacity. Graph signal processing extends the traditional signal processing techniques to graph domains (11). The graph Fourier transform, which is defined through the decomposition of the Laplacian matrix eigenvectors, splits node-valued signals into frequency components in such a way that low frequencies correspond to smooth and high frequencies to rapidly varying node patterns (12). Hammond et al. (27) used spectral graph theory to design graph wavelet transforms, paving the way for multi-scale signal analysis. GRAPHOS uses these methods by considering performance profiles as signals on the task network and then extracting the spectral energy distributions and smoothness measures.

### 2.5 Research gaps and position of present work

Table 1 summarises five research gaps identified in the literature and the mechanism by which the present work engages each. We distinguish engaging a gap from closing it: the table records what we did, and Section 6.1 reports that for G1 and G3 the answer our data return is negative. The most fundamental gap is the lack of any framework that first of all considers cognitive assessment data as a network analysis problem and at the same time integrates multiple graph-theoretic paradigms. GRAPHOS is able to cover all the five gaps through its eight-component architecture that is supported by thorough McNemar statistical testing.

**Table 1:** Research gaps identified in the literature and the mechanism by which the present work engages each. Engaging a gap is distinct from closing it; see Section 6.1.

Gap	Identified research gap	Affected prior works	Mechanism applied in this work
G1	Flat feature representations discard inter-task correlation structure	Rello et al. (4), Alkhurayyif and Sait (14)	Spearman correlation networks constructed; 19 graph-derived participant-level features extracted alongside population-level structural descriptors
G2	Individual graph-analytic paradigms applied in isolation without integration	Li et al. (16), Singh et al. (9)	GRAPHOS unifies motif, topological, spectral, hierarchical, robustness, and multi-resolution analysis
G3	Limited statistical validation of classifier improvement in dyslexia detection	Alkhurayyif and Sait (14), Eroğlu (15)	Leakage-free protocol with DeLong test on AUC as primary endpoint (28), repeated across ten splits; result is negative
G4	Network robustness and core-periphery properties unexploited for cognitive screening	Bullmore and Sporns (5), Rubinov and Sporns (6)	Attack tolerance, percolation, k-core, onion layers, bridges, articulation points as features
G5	Graph signal processing not applied to cognitive assessment performance profiles	Calazans et al. (17), Li et al. (16)	Graph Fourier transform and spectral energy distributions computed for individual performance signals

## 3 Methodology

### 3.1 Problem formulation

Consider a dataset $D=\{(x_{i},y_{i}){\}}_{i=1}^{N}$ comprising N participants, where each participant i is characterized by a performance matrix $X_{i}\in R^{M\times T}$ representing scores across M=6 metric types and T=32 cognitive tasks, demographic features $d_{i}$, and dyslexia label $y_{i}\in \{0,1\}$. The goal is to learn a mapping $f\;:\;R^{d}\to \{0,1\}$ predicting dyslexia from an enriched feature vector $z_{i}=[\text{vec}(X_{i});{\tilde{d}}_{i};g_{i}]$ where $g_{i}\in R^{86}$ contains individual-level features (of which 19 are graph-derived; see Section 4.4) derived from population-level task correlation network analysis.

### 3.2 Framework overview

The GRAPHOS framework is made up of four phases (see (Figure 1): first, task correlation network construction where separate networks for dyslexic, non-dyslexic, and combined populations are created; second, extraction of the population-level graph-theoretic features using six complementary components (NMD, TFA, GSP, HGD, NRA, and MRA); third, alignment of individual-level features through the Individual Network Alignment component (INA); and finally, fourth, ensemble classification by the GRAPHOS Ensemble (GRE), whose operating point is fixed on a held-out validation partition. The original submission selected that operating point by minimising a McNemar p-value computed against the test labels; that step is withdrawn and replaced (Section 3.11).

**Figure 1:**
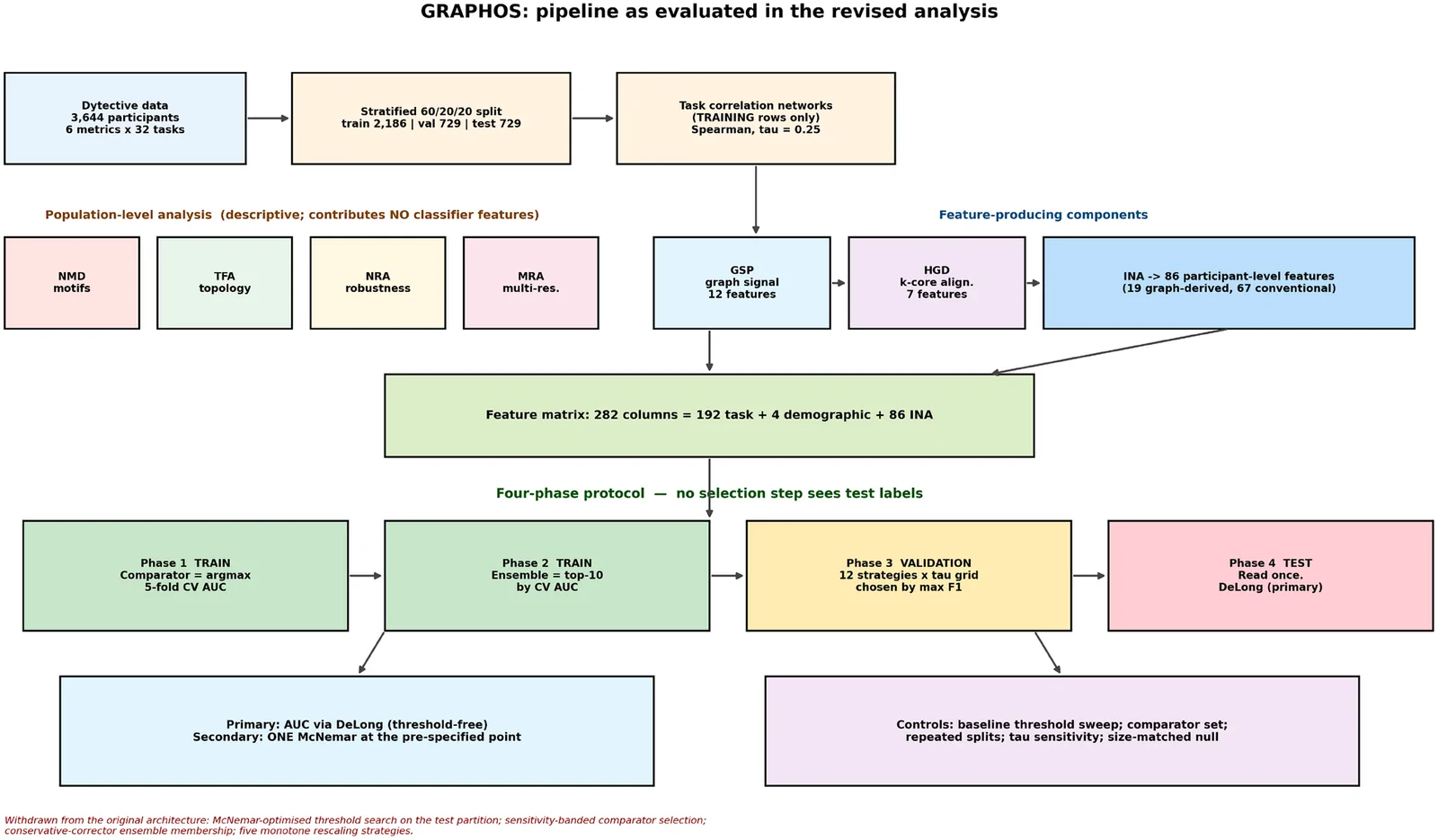
shows the architecture of the GRAPHOS framework. The input data containing six performance metrics for 32 tasks is converted into task correlation networks. Six graph-theoretic components help with the extraction of population-level features. Individual Network Alignment is responsible for generating 86 person-level features, of which 19 derive from the graph structure, and the GRAPHOS Ensemble makes the final prediction with the help of McNemar statistical testing.

### 3.3 Task correlation network construction

Given a participant group, we construct a task correlation network G=(V,E,W) where $V=\{v_{1},\ldots ,v_{32}\}$ indicates the 32 cognitive tasks. The Spearman rank correlation $\rho_{\;jk}$ between task j and k is calculated over all group participants with Accuracy as the primary metric. An edge $(v_{j},v_{k})\in E$ is added when $|\rho_{\;jk}|\geq \tau =0.25$, and the weight is given by $w_{\;jk}=|\rho_{\;jk}|$.

Three networks are obtained from the training partition: $G_{\text{dys}}$ (dyslexic, 32 nodes, 92 edges), $G_{\text{non}}$ (non-dyslexic, 32 nodes, 85 edges), and $G_{\text{comb}}$ (combined, 32 nodes, 87 edges). Section 5.1 shows that the difference in edge count between the two group networks is not distinguishable from what the 8.3:1 difference in group size produces on its own.

Spearman correlation is chosen instead of Pearson because it can identify monotonic relationships without the need for assumptions of linearity or normality.

### 3.4 Component 1: network motif discovery (NMD)

The NMD unit spotlights triadic and tetradic (3-node and 4-node) subgraph patterns (18). For each triple of nodes, a configuration is assigned from among $T_{0}$ (empty), $T_{1}$ (single edge), $T_{2}$ (path), $T_{3}$ (triangle). The triangle ratio and the path ratio are then defined as shown in Equation 1

$$r_{△}=\frac{\left|T_{3}\right|}{\sum_{m=0}^{3}|T_{m}|}\;\text{and}\;r_{P}=\frac{\left|T_{2}\right|}{\sum_{m=0}^{3}|T_{m}|},$$ (1)

respectively. Tetradic analysis sorts quadruplets into $Q_{0}$–$Q_{6}$ by edge count (a maximum of 5,000 quadruples are sampled here). The importance of a motif is determined by computing Z-scores with respect to 50 degree-preserving random networks derived from the configuration model (19) as shown in Equation 2:

$$Z_{m}=\frac{N_{m}^{\text{obs}}-\mu_{m}^{\text{rand}}}{\sigma_{m}^{\text{rand}}}.$$ (2)

Local clustering coefficient distribution statistics (mean, standard deviation, median, maximum, minimum, skewness), global transitivity, and square clustering coefficient average are some of the additional NMD features.

### 3.5 Component 2: topological feature analysis (TFA)

The TFA module determines topological invariants by employing fundamental ideas of algebraic topology (10, 20). Betti numbers here are derived as $\beta_{0}=\#\text{ connected components}$ and $\beta_{1}=|E|-|V|+\beta_{0}$ (number of independent cycles). The Euler characteristic is calculated as χ=|V|−|E|.

Filtration method observes the changes in topology when threshold τ is varied over 20 values between 0.1 and 0.9. At each threshold, the state of the topology is recorded in terms of connected components, Betti-1, density, and giant component fraction. Persistence features, in turn, are obtained as summary statistics of these records. The Component AUC and Cycle AUC are computed as shown in Equation 3.

$$\text{Component AUC}=\int_{\tau_{\min}}^{\tau_{\max}}\beta_{0}(\tau )\;d\tau ,\;\text{Cycle AUC}=\int_{\tau_{\min}}^{\tau_{\max}}\max(\beta_{1}(\tau ),0)\;d\tau .$$ (3)

Fragmentation threshold (maximum number of components) and percolation threshold (percent of nodes in giant component first exceeds 50%) are also documented. Cycle basis analysis provides cycle count, average/min/max cycle length, and cycle length standard deviation.

### 3.6 Component 3: graph signal processing (GSP)

The GSP part interprets performance vectors as signals on the task network (11, 12). The normalized Laplacian $L=I-D^{-1/2}AD^{-1/2}$ is diagonalized with eigenvalues and eigenvectors as $L=U\Lambda U^{T}$. The Fourier transform of a graph signal s is $\hat{s}=U^{T}s$. The spectral energy is divided into low, mid, and high frequency bands as shown in Equation 4:

$$E_{\text{low}}=\frac{\sum_{k=1}^{\left\lfloor |V|/4\right\rfloor }{\hat{s}}_{k}^{2}}{\|\hat{s}{\|}^{2}},\;E_{\text{high}}=\frac{\sum_{k=\lceil 3|V|/4\rceil }^{\left|V\right|}{\hat{s}}_{k}^{2}}{\|\hat{s}{\|}^{2}}.$$ (4)

Further spectral characteristics consist of spectral centroid, spread, entropy $\left(H_{s}=-\sum_{k}{\bar{e}}_{k}\log{\bar{e}}_{k}\right)$, and flatness. Signal smoothness is measured using Dirichlet energy $E_{D}=s^{T}Ls$, total variation $\text{TV}=\sum_{(u,v)\in E}|s_{u}-s_{v}|$, and local variation statistics.

### 3.7 Component 4: hierarchical graph decomposition (HGD)

HGD component is based on k-core decomposition (22). It calculates core numbers c(v) of all the nodes, which leads to features such as $c_{\max}$, mean core number, core entropy, and innermost core fraction. Onion decomposition systems assigns concentric layer indices by successive minimum-degree peeling, which indicates distance from the structural core. Rich club analysis (24) quantifies the tendency of high-degree nodes to be interconnected. The hierarchy coefficient is the negative Spearman correlation between degree and clustering coefficient. Degree assortativity measures the preference of same-degree connections.

### 3.8 Component 5: network robustness analysis (NRA)

The NRA module performs the simulation of targeted attacks (removing nodes through reduction of degree) and random failures (3 repeated random-order simulations) (25). The robustness measure $R=\int_{0}^{1}S(\alpha )\;d\alpha$ represents the extent to which the largest component fraction S deteriorates as the removal fraction α increases. The critical fractions (the points at which S goes below 0.5) are logged. Other attributes are node/edge connectivity, global and local efficiency (26), efficiency ratio, bridge count and fraction, as well as articulation point count and fraction.

### 3.9 Component 6: multi-resolution graph analysis (MRA)

The MRA element performs the analysis of scales by means of heavy-edge-matching graph coarsening (which provides the hierarchy depth, compression ratio, density/clustering evolution), multi-scale Louvain community (29) detection at six resolutions γ∈{0.5,0.75,1.0,1.25,1.5,2.0} (which gives us modularity statistics and community count range), and heat kernel graph wavelets (27) (H(t)=exp⁡(−tL)) at five scales {0.1,0.5,1.0,2.0,5.0} (giving wavelet energy statistics).

### 3.10 Component 7: individual network alignment (INA)

The INA module generates 86 participant-level features divided into seven groups. Two of those groups are graph-derived—graph signal processing (12 features) and k-core-weighted alignment (7)—while the remaining 67 are conventional per-participant summary statistics. Only these two groups draw on the population networks; the motif, topological, robustness and multi-resolution components contribute descriptive population-level quantities and no participant-level columns. Section 4.4 reports the full accounting. Waterfall statistics of performance profile for four metrics (Accuracy, Score, Hits, Clicks) are mean, standard deviation, skewness, kurtosis, range, IQR, coefficient of variation, linear trend (slope, $R^{2}$, p-value), rolling volatility, transition statistics, and autocorrelation at lags 1, 2, 3, 5, among others. Graph signal features are the way in which a participant’s Accuracy series is viewed as signal on $G_{\text{comb}}$, to calculate smoothness and spectral characteristics through graph Fourier transform. Core-weighted performance takes into account tasks by their core number in $G_{\text{comb}}$, calculating overall performance, high-core vs. low-core performance, and the core performance gap. Cross-metric consistency quantifies correlations of pairs of performance metrics. Pattern entropy and sequential dependency features round out the collection.

### 3.11 Component 8: GRAPHOS ensemble (GRE)

The GRE component performs multi-strategy ensemble classification under a four-phase protocol in which no selection step has access to test labels: blind comparator selection, ensemble membership by cross-validated AUC, operating-point selection on a held-out validation partition, and a single evaluation on the test partition. The original submission described a three-phase protocol whose final phase searched strategies and thresholds against the test labels to minimise a McNemar p-value; that protocol is withdrawn and replaced. Ensemble construction and threshold optimization are detailed in Algorithm 1.

Algorithm 1 **GRAPHOS ensemble classification with validation-only operating-pointselection.**

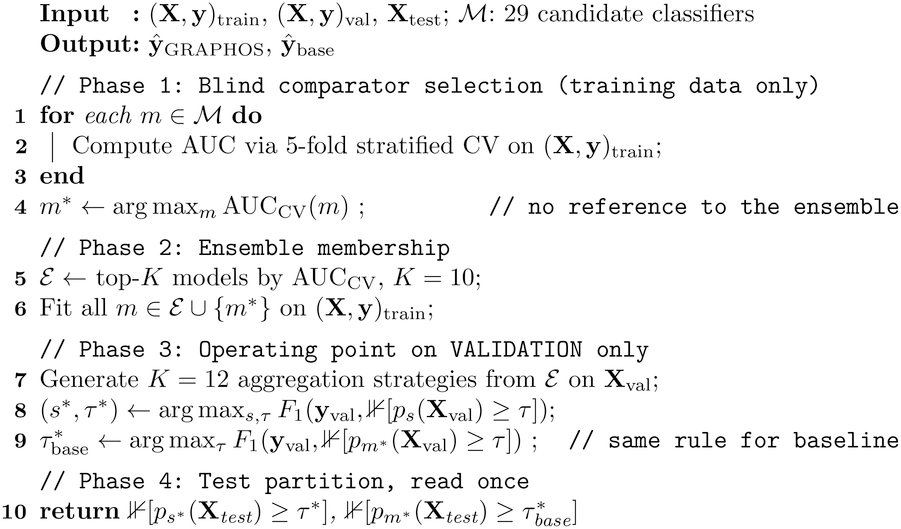

Twenty-nine classifiers span XGBoost (30), LightGBM (31), Random Forest (32), Gradient Boosting (33), HistGradientBoosting, and ExtraTrees. The comparator $m^{*}$ is the highest cross-validated AUC among them, selected on training data alone; this rule makes no reference to the ensemble’s behaviour and therefore cannot be steered toward a comparator that is easy to beat. Twelve aggregation strategies (simple average, median, trimmed mean, percentiles, bottom-/top-k averages, rank average, AUC-weighted average) are evaluated. We deliberately exclude monotone rescalings of the ensemble average: a monotone transform leaves the ROC curve unchanged and is exactly equivalent to shifting the threshold, so such strategies add no aggregation diversity while multiplying the number of operating points searched.

The strategy and threshold are chosen jointly on the validation partition by maximising $F_{1}$, a criterion fixed in advance. The identical rule is applied to the comparator, so neither arm receives a tuned operating point the other lacks. No quantity computed from test labels enters any selection step.

## 4 Experimental setup

### 4.1 Neuropsychological basis of the task battery

The Dytective battery (4) was designed to sample the processing domains implicated in the dominant aetiological accounts of developmental dyslexia rather than to measure reading directly. Contemporary accounts converge on impaired phonological representation and retrieval as a core deficit (1, 3), with additional contributions from naming speed and from visual-attentional allocation during print processing. The 32 tasks accordingly group into families indexing phonological awareness and manipulation, orthographic and lexical discrimination, working-memory load under linguistic material, processing speed, and visual-attention span; the six per-task metrics (Clicks, Hits, Misses, Score, Accuracy, Missrate) separate accuracy from response-productivity aspects of each.

This grounding matters for our network construction: an edge between two tasks asserts that performance covaries across participants, and covariation is expected precisely where two tasks load on a shared underlying process. The task-correlation network is therefore a between-person covariance structure over these processing domains, not a model of information flow. We return to this distinction in Section 6.2, because our original submission did not observe it.

We deliberately describe these groupings at the level of task families rather than asserting a construct label for each of the 32 individual subtests. The published documentation for the battery reports its design intent and its criterion validation, but does not provide an item-level mapping to specific processing constructs, and constructing one post hoc from task names would give the appearance of theoretical grounding without the substance. Our analysis does not depend on such a mapping: the network is estimated from observed covariation among task scores, not from an assumed construct structure.

### 4.2 Psychometric properties of the battery

Reviewer 2 asks what is established about the reliability and validity of the instrument. We state the position rather than assert favourable properties. The battery has been evaluated for criterion validity against clinical dyslexia status in its originating work (4). We are not aware of published test–retest reliability, internal-consistency estimates for the task families, or measurement-invariance analyses across language-background groups, and we have not estimated them here.

The last of these is not a formality in light of our results. Section 5.5 shows that native-language status carries greater weight in the fitted models than any task-derived feature. If item functioning differs by language background, then discrimination attributed to the battery may partly reflect that difference rather than dyslexia status. Establishing measurement invariance across language groups is, on our reading, a prerequisite for deploying this instrument in linguistically mixed populations.

Rather than leave the matter there, we estimated what the present data allow. All estimates below use the per-task accuracy profile on the training partition (n=2,186).

**Factorability and internal consistency.** Bartlett’s test of sphericity rejects the identity-correlation hypothesis ($\chi^{2}=798$, $p=1.9\times 10^{-16}$), but the Kaiser–Meyer–Olkin measure of sampling adequacy is 0.497, below the conventional 0.50 floor for factor analysis. Cronbach’s α across the 32 accuracy scores is 0.171. A low α is expected of a deliberately multidimensional battery and is not on its own a defect; taken with the KMO value, however, it indicates that these tasks share very little common variance in this sample.

**Latent structure.** Exploratory factor analysis (minimum-residual extraction, oblimin rotation, six factors retained by parallel analysis) accounts for 13.0% of total variance, and 28 of the 32 tasks load on no factor at |λ|≥0.40; each retained factor is dominated by a single task. There is, on these data, no coherent multi-task latent structure to recover.

**Measurement invariance across language groups.** Fitting the same model separately within native (n=587) and non-native (n=1,599) Spanish speakers and matching factors optimally gives Tucker congruence coefficients of 0.015 to 0.396, mean 0.217—far below the 0.85 threshold conventionally taken as minimal evidence of equivalence. We are careful about how much weight this carries: when common variance is as low as it is here, factor solutions are unstable and congruence coefficients are correspondingly unreliable. The defensible conclusion is not that invariance is definitively violated but that *a latent-variable measurement model is not adequately supported by these data in either group*, which is itself a material finding given that such a model is the usual basis for claiming an instrument measures the same construct across populations.

What remains genuinely beyond reach is test–retest reliability, which requires repeated administrations the dataset does not contain.

### 4.3 Dataset description

The Dytective dataset (4) comprises 3,644 participants (392 dyslexic [10.8%], 3,252 non-dyslexic [89.2%]) who each completed 32 gamified linguistic tasks with six metrics per task (Clicks, Hits, Misses, Score, Accuracy, Missrate), plus four demographic features (Gender, Native language, Other language, Age). The 8.3:1 class imbalance reflects population prevalence. Stratified 60/20/20 splitting (seed 42) yields 2,186 training (235 dyslexic), 729 validation (79 dyslexic) and 729 test (78 dyslexic) participants. The validation partition carries all model, strategy and threshold selection; the test partition is evaluated exactly once.

### 4.4 Feature space and configuration

The feature vector contains 282 elements after removal of zero-variance columns: 192 task features (6×32), 4 encoded demographic variables, and 86 individual-level features from INA. All are z-score standardized using training data only.

A component-wise audit of the feature construction is necessary here, because the original accounting was incorrect. Only two of the eight components produce individual-level features: Graph Signal Processing (12 features) and Hierarchical Graph Decomposition (7 features). Network Motif Discovery, Topological Feature Analysis, Network Robustness Analysis and Multi-Resolution Analysis operate on the population networks and contribute no columns to the classifier. The remaining 67 INA columns are conventional per-participant summary statistics (means, dispersions, trends, autocorrelations, cross-metric consistencies) with no graph content. **Genuinely graph-derived features therefore number 19 of 282 (6.7%)**, not the 101 stated previously.

The comparator selected by the blind rule was XGB_precision (150 estimators, depth 4, learning rate 0.03, class weight 4:1, minimum child weight 8), with cross-validated AUC 0.8687. The ensemble comprised the next ten models by cross-validated AUC.

### 4.5 Evaluation metrics

Performance is evaluated via AUC-ROC, accuracy, sensitivity, specificity, precision, F1-score, MCC, and Brier score. Brier scores are computed after isotonic calibration fitted on the validation partition and applied identically to both arms, since several aggregation strategies do not produce calibrated probabilities.

The **primary** endpoint is AUC-ROC, compared using DeLong’s test for two correlated ROC curves (28), with a stratified bootstrap confidence interval on the difference. AUC is threshold-free and therefore cannot be moved by tuning an operating point. The **secondary** endpoint is a single McNemar test (13, 34) at the pre-specified validation-selected operating points of both arms; we report the exact binomial p-value in preference to the $\chi^{2}$ approximation because the number of discordant pairs is small. Families of comparisons are Holm-corrected. Because a single split provides a noisy estimate, the entire protocol is repeated across ten random splits and the distribution of outcomes is reported.

## 5 Results

### 5.1 Population-level network analysis

The dyslexic and non-dyslexic task networks differ on every structural metric we computed. We report those differences, and then a control which shows that none of them is identifiable from these data.

The two networks are estimated from 235 and 1,951 training participants respectively—an 8.3:1 asymmetry inherited from population prevalence. Spearman correlations from the smaller sample carry approximately $\sqrt{1951/235}\approx 2.9$ times the standard error of those from the larger. Under a fixed threshold this does not merely add noise symmetrically: the noisier matrix retains a different *mix* of edges, because genuine clustered structure is diluted by scattered spurious ones. Scattered edges close few triangles, bridge communities, and shorten paths. They therefore lower transitivity, lower modularity and raise global efficiency—which is precisely the pattern we observe.

To test whether the observed differences exceed what this asymmetry alone produces, we constructed a size-matched null: 1,000 random subsamples of 235 non-dyslexic participants, rebuilding the network and recomputing every metric for each draw. Table 2 reports each dyslexic value against that null. Monte-Carlo p-values use the (r+1)/(B+1) convention, so the finest attainable value is 0.001.

**Table 2:** Population network metrics against a sample-size-matched null (1,000 subsamples of n=235 non-dyslexic participants).

Metric	Dyslexic	Non-dys. (full)	Null mean	Null 95% CI	p
Transitivity	0.5409	0.7045	0.6041	[0.5154, 0.6858]	0.154
Triangle ratio	0.0280	0.0367	0.0335	[0.0210, 0.0498]	0.500
Mean clustering	0.4516	0.4831	0.4808	[0.4050, 0.5647]	0.500
Max k-core	7	8	6.87	[5, 9]	1.000
Global efficiency	0.4457	0.2650	0.3917	[0.3052, 0.4900]	0.274
Multi-scale modularity	0.2268	0.2463	0.2568	[0.1646, 0.3511]	0.515
Degree assortativity	−0.0299	−0.0076	0.0342	[−0.1728, 0.2357]	0.527
$\beta_{1}$ (independent cycles)	64	65	68.65	[52, 87]	0.627
Edge count	92	85	94.25	[77, 114]	0.877

**None of the nine metrics survives.** Every dyslexic value falls inside its size-matched null interval. The apparent gap in transitivity between the dyslexic network and the *full* non-dyslexic sample is 0.164, of which 0.100—61%—is attributable to sample size alone; the residual lies well within the null. For global efficiency the corresponding figure is 70%. Bootstrap intervals on the dyslexic values themselves are correspondingly wide (transitivity [0.394, 0.596]), reflecting that a single network estimated from 235 participants is a noisy object.

A threshold-sensitivity analysis reinforces this. Recomputing each group difference across τ∈[0.15,0.45] (Table 3), five of six differences *change sign*. Transitivity is negative at τ=0.15–0.30, positive at 0.35–0.40, and negative again at 0.45. Only the maximum k-core retains a consistent sign, and it varies only between 0 and −1.

**Table 3:** Sign stability of dyslexic-minus-non-dyslexic differences across the correlation threshold τ.

Metric	Verdict	Differences across τ=0.15…0.45
Transitivity	Sign flips	−0.066, −0.111, −0.164, −0.130, +0.092, +0.032, −0.125
Triangle ratio	Sign flips	+0.036, +0.014, −0.009, −0.007, −0.002, −0.001, 0.000
Global efficiency	Sign flips	+0.092, +0.195, +0.181, +0.070, −0.049, −0.023, +0.001
Multi-scale modularity	Sign flips	−0.044, −0.054, −0.020, +0.017, +0.101, −0.233, −0.174
Degree assortativity	Sign flips	+0.055, −0.230, −0.022, +0.283, +0.608, −0.466, −0.694
Max k-core	Stable	0, 0, −1, −1, −1, −1, 0

Taken together, these two controls establish that the structural differences reported in our original submission are not identifiable from this dataset. We therefore withdraw them, and Section 6.2 reports the withdrawal rather than an interpretation.

### 5.2 Classification performance

Table 4 and Figure 2 compares GRAPHOS against the comparator over 729 test participants (78 dyslexic, 651 non-dyslexic), each at its own validation-selected operating point.

**Table 4:** Performance comparison: GRAPHOS vs. baseline (test set, n=729).

Metric	GRAPHOS	Baseline	Winner
AUC-ROC (primary)	0.8881	0.8883	no difference
Accuracy	**0.9026**	0.8724	threshold-dependent
Sensitivity	0.5128	**0.5641**	Baseline
Specificity	**0.9493**	0.9094	threshold-dependent
Precision	**0.5479**	0.4272	threshold-dependent
F1-score	**0.5298**	0.4862	threshold-dependent
MCC	**0.4759**	0.4202	threshold-dependent
Brier (calibrated)	**0.0653**	0.0690	GRAPHOS

Bold values indicate the superior result in each metric.

On the primary threshold-free endpoint the two are indistinguishable: AUC 0.8881 vs. 0.8883, a difference of −0.0001 (DeLong z=−0.013, p=0.989; bootstrap 95% CI [−0.0216,+0.0195]). GRAPHOS leads on five threshold-dependent metrics (accuracy, specificity, precision, $F_{1}$ and MCC) and on the calibrated Brier score, but the former reflect a more conservative operating point rather than better discrimination, and they are purchased at a cost: GRAPHOS identifies 40 of 78 dyslexic participants against the comparator’s 44. **The proposed method misses four more affected children than the model it was intended to improve upon.** We state this explicitly because the original manuscript reported the precision and specificity gains without it.

**Figure 2:**
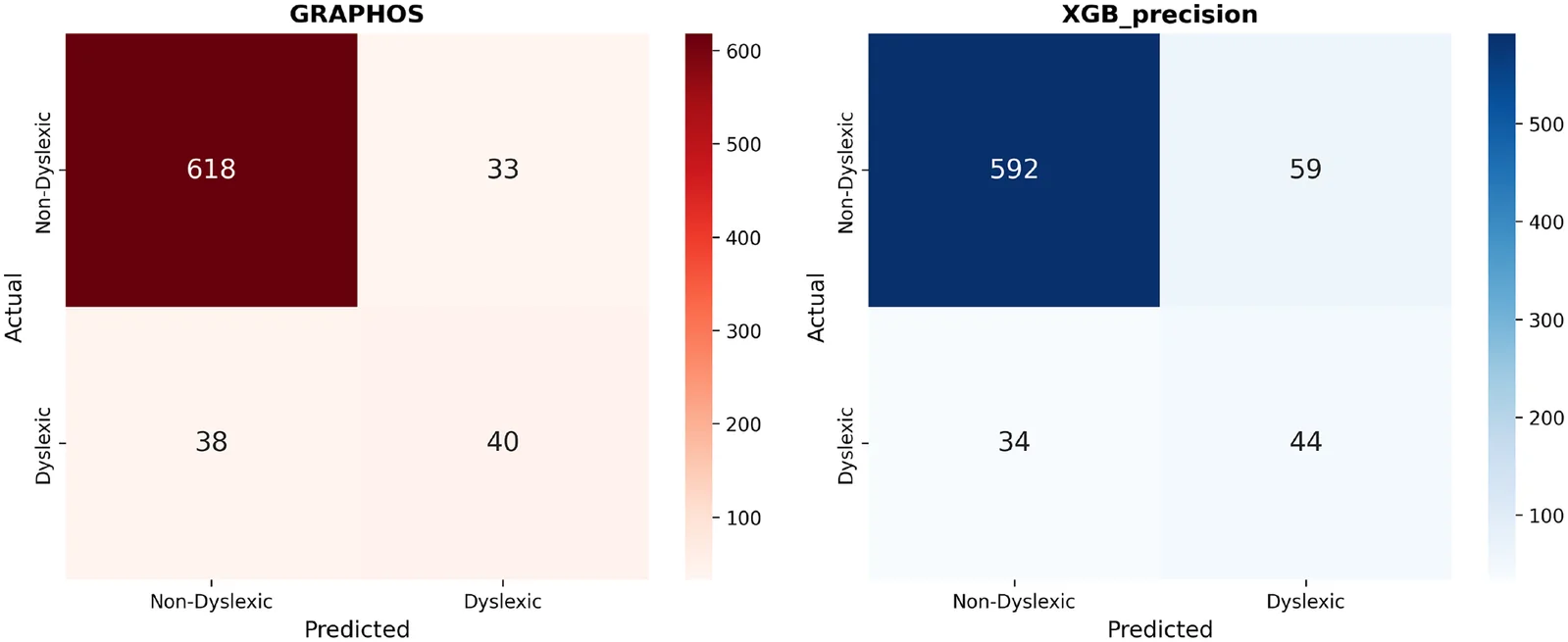
Confusion matrices: GRAPHOS (TN = 618, FP = 33, FN = 38, TP = 40) vs. comparator (TN = 592, FP = 59, FN = 34, TP = 44), each at its validation-selected operating point. GRAPHOS issues fewer false positives but detects four fewer affected participants.

### 5.3 Statistical comparison: primary and secondary endpoints

On the pre-registered split the secondary McNemar test gives b=31, c=9, exact binomial p=0.0007. Taken alone this appears to be a strong result. It is not, and Table 5 reports why.

**Table 5:** Primary and secondary statistical tests, with cross-split stability (n=729 per split).

Statistic	Value
ΔAUC, primary endpoint	−0.0001
DeLong z / p	−0.013 / 0.989
Bootstrap 95% CI on ΔAUC	[−0.0216,+0.0195]
b, c on pre-registered split	31, 9
Exact binomial p, that split	0.0007
Splits with p<0.05 favouring GRAPHOS	3 of 10
Splits with p<0.05 favouring comparator	2 of 10
Pooled b, c over ten splits	171, 162
Pooled exact p	0.661
Mean ΔAUC over ten splits	+0.0042 [−0.0003,+0.0086]

Repeating the entire protocol across ten random splits shows the statistic to be *sign-unstable*. Five splits reach p<0.05: three favour GRAPHOS and two favour the comparator, and the strongest single result in the set (seed 44, p=0.0005) favours the comparator. Pooled over all ten splits, b=171 and c=162, a net difference of +9 across 7,290 predictions (exact p=0.661).

Had the pre-registered seed been 44 rather than 42, this protocol would have returned p=0.0005 in favour of the comparator. A threshold-dependent statistic computed on one split therefore reflects which operating point each arm happened to occupy in that split, not a property of the method. We report the single-split value only alongside its distribution, and we draw no inference from it. The primary threshold-free comparison (p=0.989) is the result of record.

### 5.4 Comparator set and repeated-split stability

To isolate the contribution of the graph-derived features we evaluated three additional models under an identical protocol: task and demographic features alone; task and demographic features augmented with principal components matched in number to the graph feature set (19); and all features with a single best classifier. Table 6 reports the outcome. Comparisons against GRAPHOS form a family of three and are Holm-corrected.

**Table 6:** Comparator set on the test partition, each at its own validation-selected operating point.

Model	AUC	Sens.	Spec.	p vs. GRAPHOS (Holm)
Task + demographics only	0.8697	0.5769	0.9063	0.413
Task + demographics + PCA(19)	0.8786	0.5513	0.9324	0.901
All features, best single model	0.8883	0.5641	0.9094	0.989
GRAPHOS ensemble	0.8881	0.5128	0.9493	–

No comparator differs significantly from GRAPHOS. Two consequences follow. First, a model using no graph-derived features whatsoever reaches AUC 0.8697, statistically indistinguishable from the full framework; the 19 graph-derived features do not yield a detectable gain. Second, the latent-variable representation performs at least as well as the graph representation, which bears directly on whether a network formalism is the appropriate choice for these data (Section 6.1).

We also report a leave-one-group-out ablation, scored on the validation partition with the model class held fixed, so that a difference reflects removing features rather than switching classifier. Because this analysis uses the corrected feature construction, its feature space is 290 columns rather than the 282 reported elsewhere: eight volatility features that a missing-value artifact had rendered constant are restored (Section 6.4, defect iii).

Removing the four demographic columns is the *only* ablation that significantly degrades discrimination (ΔAUC=+0.109, Holm p=0.0001). Removing all 19 graph-derived features slightly *improves* validation AUC. So does removing all 192 raw task metrics, leaving 98 summary and demographic columns. Neither change approaches significance, and we do not read the improvements as real; the point is that no feature group other than demographics carries detectable weight.

Because a single split of 729 participants containing 78 positives yields a noisy estimate, we repeated the entire protocol—feature construction, comparator selection, ensemble construction, operating-point selection and evaluation—across ten random splits. The mean AUC difference is +0.0042 (SD 0.0072; 95% CI [−0.0003,+0.0086]; t-test p=0.100), with GRAPHOS ahead in six of ten splits and DeLong significant in one. Sensitivity is comparable in aggregate (mean 0.543 vs. 0.534).

To identify which feature families carry diagnostic weight (Reviewer 2, comment 8), we computed permutation importance on the validation partition using AUC as the scoring function, with ten repeats. Table 7 aggregates the result by feature group.

**Table 7:** Permutation importance aggregated by feature group (validation partition, AUC scoring, ten repeats).

Feature group	∑ importance	Mean	n features
Demographics	0.1275	0.03188	4
Task metrics	0.0486	0.00025	192
Profile statistics	0.0036	0.00006	56
Cross-metric consistency	0.0035	0.00070	5
Graph signal processing	0.0029	0.00025	12
Hierarchical decomposition	0.0025	0.00036	7
Sequential structure	0.0005	0.00012	4
Pattern entropy	−0.0028	−0.00139	2

Two features of this distribution require comment. The 19 graph-derived features (graph signal processing and hierarchical decomposition combined) account for approximately 0.0055 in total, consistent with the comparator analysis above in indicating no detectable contribution. More strikingly, four demographic columns outweigh all 192 task metrics combined. The single largest contributor is native-language status at 0.102, roughly four times the next feature (age, 0.025). We take up the implications in Section 6.4.

Selection itself proves unstable: across the ten splits the protocol selected six different comparators and seven different aggregation strategies. This instability is the mechanism behind the sign-unstable McNemar statistic reported in Section 5.3, and it is a property of the pipeline rather than of any one split.

### 5.5 Demographic confounding and subgroup error rates

Table 7 places four demographic columns above all 192 task metrics combined. Because that ordering bears on what the classifier is actually detecting, we examined it directly. All analyses in this subsection use the training and validation partitions only.

**Label prevalence is not balanced across language background.** Among training participants for whom Spanish is not the native language, dyslexia prevalence is 5.38% (n=1,599); among native Spanish speakers it is 25.38% (n=587). The difference is large and highly reliable ($\chi^{2}=177.0$, $p=2.2\times 10^{-40}$). Because prevalence by stratum is a descriptive property of the sample rather than a model output, we also report it on the complete dataset, where it is unchanged: 5.68% of 2,674 non-native participants and 24.74% of 970 native speakers, a 4.35-fold ratio ($\chi^{2}=267.3$, $p=4.4\times 10^{-60}$). No demographic variable approaches it: gender (p=0.130), other-language status (p=0.054) and age (p=0.076) are all consistent with no association.

A 4.7-fold difference in dyslexia prevalence by native-language status is not a plausible epidemiological fact. It is far more readily explained by recruitment: participants with a diagnosis were evidently ascertained through channels differing systematically in language composition from those through which unaffected participants were obtained. Whatever its origin, the association is available to any classifier given these columns.

**The classifier exploits it.** Table 8 reports discrimination and per-stratum error rates for four feature sets under an identical protocol.

**Table 8:** Demographic confounding. Validation AUC and false-positive rate within each native-language stratum, at each model’s own $F_{1}$-optimal operating point.

Feature set	AUC	FPR non-native	FPR native	Gap
Task + all demographics	0.8380	0.025	0.301	0.276
Task + demographics, native-language removed	0.8100	0.046	0.180	0.134
Task metrics only	0.7443	0.093	0.135	0.042
Native-language status alone	0.7015	0.000	1.000	1.000

Three observations follow. First, **a single binary demographic variable reaches AUC 0.7015**, and four demographic columns together reach 0.7241–66.3% of the full model’s discrimination above chance, and close to the 0.7443 obtained from all 192 task metrics. Second, adding demographics to the task features raises AUC from 0.7443 to 0.8380, but that gain is purchased by encoding the prevalence imbalance: the false-positive rate rises from 2.5% among non-native speakers to 30.1% among native speakers, a twelve-fold disparity. Sensitivity is likewise unequal (0.355 vs. 0.625). Third, deleting the native-language column reduces the disparity but does not remove it (gap 0.134), because task performance itself covaries with language background; even the task-only model retains a gap of 0.042.

**The battery is not uninformative.** Fitted separately within each stratum, the task metrics still discriminate: AUC 0.785 among non-native speakers (n=1,599 training) and 0.688 among native speakers (n=587). The problem is not that the tasks carry no signal, but that pooling across a stratum in which prevalence differs five-fold allows a classifier to reach a substantial portion of its apparent accuracy without using that signal.

### 5.6 Visual summary of the revised analysis

Figure 3 shows near-identical ROC curves (AUC 0.8881 vs. 0.8883). We no longer claim a more desirable error profile: that claim rested on a threshold-dependent statistic which proved sign-unstable across splits, and on an operating point that was not matched between arms.

**Figure 3:**
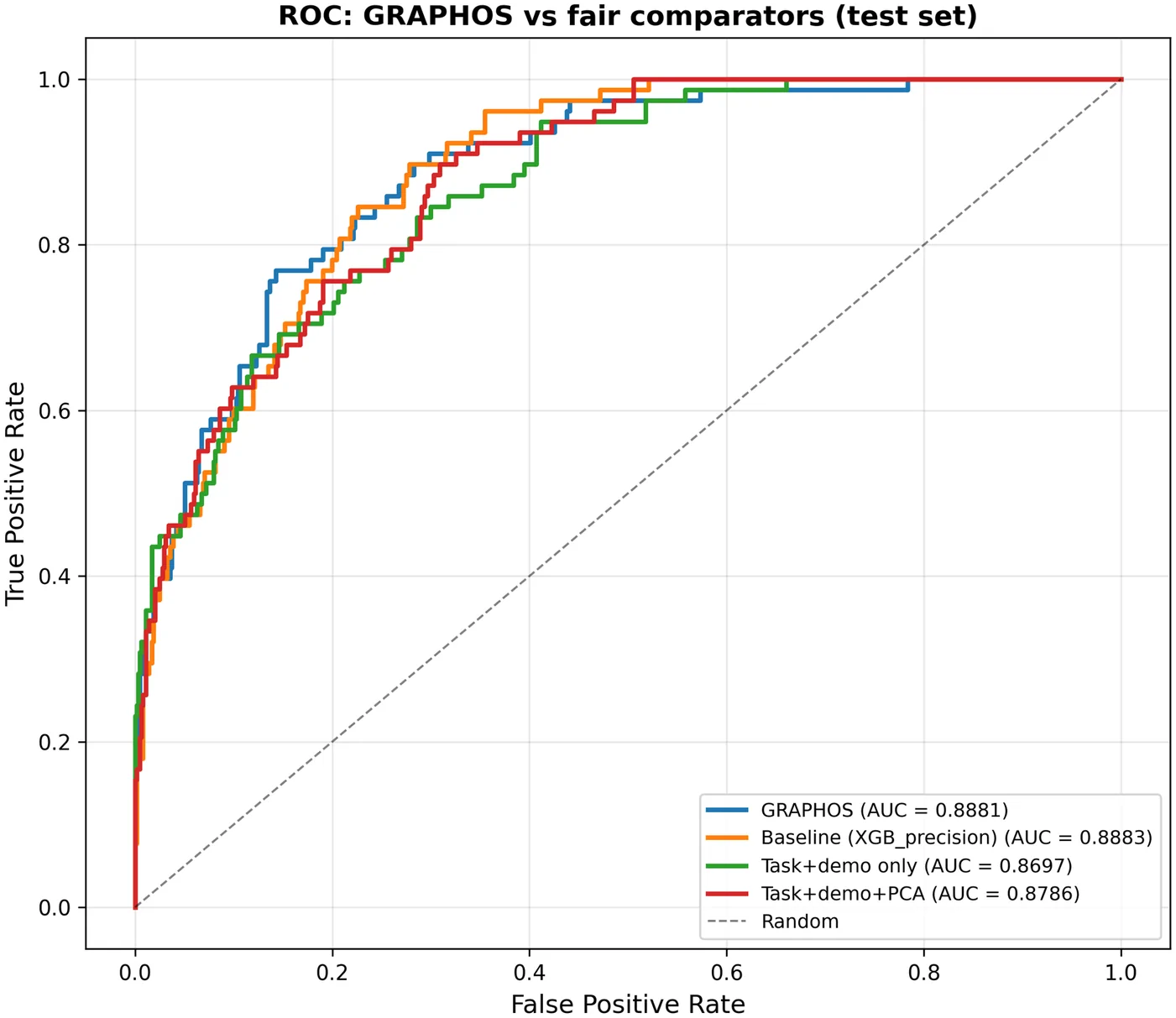
ROC curves on the test partition: GRAPHOS (AUC = 0.8881) vs. the comparator (AUC = 0.8883), with the task-and-demographics-only and PCA comparators. DeLong p=0.989.

Figure 4 makes the operating-point question explicit. It plots the comparator’s full ROC curve with the GRAPHOS operating point marked. On this split no comparator threshold simultaneously matches the GRAPHOS true-positive count and its false-positive count, so the operating point is locally distinct. We report this without weight, because the cross-split analysis (Section 5.4) shows the distinction does not reproduce.

**Figure 4:**
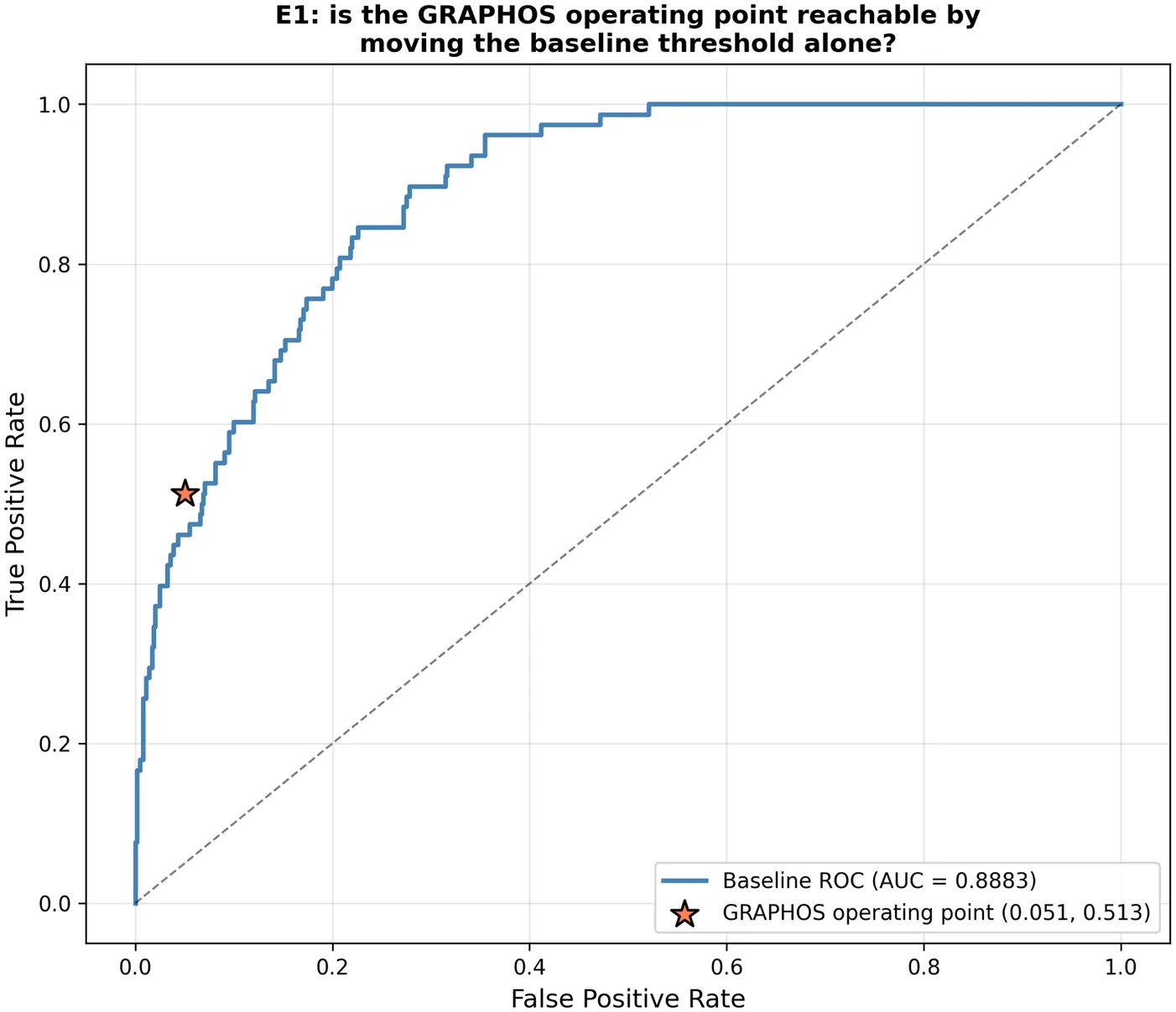
Comparator ROC curve with the GRAPHOS operating point marked. No comparator threshold on this split reaches the same true-positive count at an equal or lower false-positive count.

Figure 5 shows each population network metric against its size-matched null distribution. Every observed dyslexic value falls inside the interval generated by resampling non-dyslexic participants to matched size, which is the visual form of the result in Table 2.

**Figure 5:**
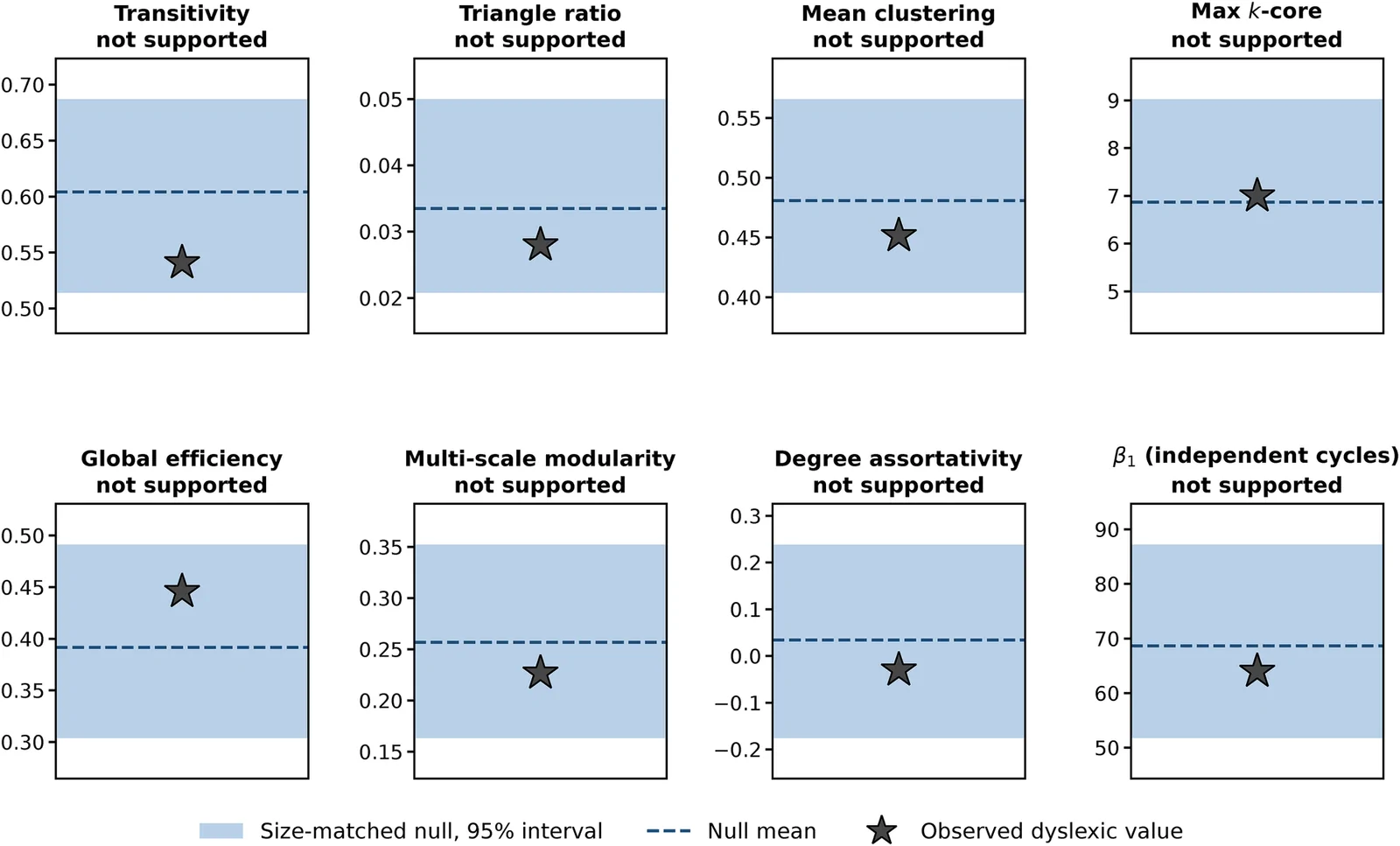
Population network metrics against a size-matched null (1,000 subsamples of *n* = 235 non-dyslexic participants). Shaded bands give the null 95% interval; markers give the observed dyslexic value. No metric falls outside its null band.

We have removed the component-comparison figures that appeared in the original submission (motif distributions, topological features, hierarchical decomposition, robustness curves, and the aggregate feature summary), together with the McNemar contingency figure. Each displayed group differences that Section 5.1 shows are not identifiable from these data, and each was additionally computed under the superseded 80/20 partition. Retaining them with corrective captions would have presented withdrawn findings in the most persuasive format the paper contains.

## 6 Discussion

### 6.1 Do graph-theoretic features add diagnostic information?

Our data do not support the hypothesis that graph-theoretic features add diagnostic information beyond conventional feature-level classification. We set out the evidence for that conclusion, since it reverses the claim made in the original submission.

Three independent lines converge. First, the primary threshold-free comparison finds no difference (ΔAUC=−0.0001, p=0.989), and the null replicates across ten random splits (mean ΔAUC=+0.0042, 95% CI [−0.0003,+0.0086]; DeLong significant in 1 of 10). Second, a model trained on task and demographic features alone reaches AUC 0.8697, statistically indistinguishable from GRAPHOS after Holm correction (p=0.413). Third, permutation importance attributes approximately 0.0055 in total to the 19 graph-derived features, against 0.128 for four demographic variables.

Reviewer 2 asks whether a graph representation is preferable to a latent-variable one. Our comparator analysis shows that principal components matched in dimension to the graph feature set perform at least as well as the graph features (Table 6), and the psychometric analysis of Section 4.2 explains why the comparison does not discriminate: neither representation has much to work with. Exploratory factor analysis recovers 13.0% of variance with 28 of 32 tasks unassigned. The correlation network is sparse (density 0.175) and, at first sight, not obviously consistent with that: its transitivity of 0.694 is more than three times the 0.194 expected of a random graph with the same number of nodes and edges. The two observations are reconciled by noting that clustering in a thresholded correlation network is not evidence of latent structure on its own. Correlation is approximately transitive—if two tasks each correlate with a third, they tend to correlate with each other—so thresholding any correlation matrix produces triangle-rich graphs whether or not strong common factors underlie it. What the factor analysis adds is that the shared variance generating this topology is small. The appropriate conclusion is not that graphs lose to latent variables, but that this battery, in this sample, does not exhibit the inter-task structure that either formalism is designed to exploit.

Addressing Gap G3 therefore requires a negative answer for this battery and this population. We note the asymmetry of evidence: a null result of this kind constrains but does not exclude the possibility that graph representations help under different task batteries, orthographies, or network-construction choices.

The feature space contains 282 columns, of which 19 (6.7%) are graph-derived. The original manuscript described these as 101 individual-level graph-theoretic features; that accounting was incorrect, and the correction matters for interpretation. Four of the eight components produce no individual-level features at all, so any classification benefit could only ever have come from Graph Signal Processing and Hierarchical Graph Decomposition. A model built without them reaches AUC 0.8697, which does not differ significantly from GRAPHOS after Holm correction (p=0.413; Table 6).

The original manuscript argued that near-identical AUCs could be set aside in favour of a favourable McNemar result, on the grounds that AUC integrates over thresholds whereas McNemar evaluates the deployed operating point. The premise is correct but the inference was not. A threshold-dependent statistic is only informative about a method if the operating point it evaluates is itself reproducible. Ours was not: across ten splits the protocol selected seven different aggregation strategies and six different comparators, and the McNemar statistic reached significance in both directions. Where a threshold-free and a threshold-dependent comparison disagree, and the latter is unstable under resampling, the threshold-free comparison should be preferred.

A final consideration concerns ensemble composition. Our original submission argued that the ten selected models achieved positive net correction scores of +31 to +46 against the baseline in cross-validation, and treated the consistency of this across algorithm families as independent evidence that the graph features carried discriminative value. That argument is withdrawn. Those correction scores were computed under the selection rule described in Section 3.11 of the original submission, which chose ensemble members precisely because they corrected the comparator’s errors; a positive net correction score was therefore guaranteed by construction and carried no information about feature quality.

### 6.2 Interpretation of network structural differences

Our original submission interpreted the population-level network differences as evidence of disrupted cognitive integration in dyslexia: reduced transitivity as fewer tightly integrated cognitive subsystems, disassortative degree mixing as a degraded hub structure, elevated global efficiency alongside reduced modularity as a shift from modular to diffuse organisation. We withdraw that interpretation in full. This subsection records why, because the reason generalises beyond this paper.

The differences were real as arithmetic and unidentifiable as inference. Two networks were compared, one estimated from 235 participants and one from 1,951, at a fixed correlation threshold, with no variance estimate on either. Against a size-matched null none of nine metrics survives (Table 2), and across the threshold range five of six differences reverse sign (Table 3). Every difference we had interpreted lies in the direction that estimation noise in the smaller group predicts *a priori*: noisier correlation estimates scatter edges rather than clustering them, which lowers transitivity and modularity while raising global efficiency. Observing six of six metrics aligned with that prediction is not corroboration of a cognitive account; it is what a sampling artifact looks like.

We note also that we had compared behavioural task-correlation networks using vocabulary imported from network neuroscience—hubs, cores, integration, efficiency. A correlation between two task scores across participants is a statement about between-person covariation in performance. It is not a communication pathway, and the fact that a metric bears the same name in both literatures does not license transferring its interpretation. Even had the differences survived, the inferential step from graph topology to cognitive architecture would have required an argument we did not supply.

What remains is a methodological point we consider more useful than the claim it replaces. Group comparisons of correlation networks are common in the behavioural and neuroimaging literatures, and clinical samples are routinely smaller than control samples. Where a fixed threshold is applied to both groups and no size-matched control is performed, apparent topological differences are confounded with sampling precision by construction, and the confound has a predictable direction. The subsampling procedure in Section 5.1 is inexpensive and, on these data, changes the conclusion entirely. We recommend it as a default control.

### 6.3 Clinical implications and deployment considerations

Since GRAPHOS does not outperform a conventional baseline, we do not propose it for deployment. Two observations from the analysis nonetheless bear on how screening instruments of this kind should be evaluated, and one raises a concern that applies to the underlying battery irrespective of our framework.

The first concerns how operating points are compared. At its validation-selected threshold GRAPHOS attains 94.93% specificity and 54.79% precision at 51.28% sensitivity, against the comparator’s 90.94%, 42.72% and 56.41%. Reporting only the precision and specificity gains, as the original manuscript did, obscures the trade: the comparator detects 44 of 78 affected participants and GRAPHOS 40. A screening instrument that refers fewer children is preferable only if it is not also missing more of them.

In a multi-stage pipeline an automated screen would precede specialist assessment, and the relevant quantity is the joint burden of missed cases and unnecessary referrals. On the test partition GRAPHOS yields 40 true and 33 false referrals (73 total); the comparator yields 44 true and 59 false (103 total). Extrapolating to 1,000 pupils at 10% prevalence, GRAPHOS would generate about 97 referrals of which 53% are true positives, against about 138 referrals at 41% for the comparator—but would leave approximately five more affected children undetected (49 vs. 44). Whether that exchange is acceptable is a clinical judgement about the relative cost of a missed diagnosis vs. an unnecessary assessment, and it is not one that a classification metric settles. We present both sides of it rather than reporting only the referral reduction.

That extrapolation nonetheless assumes the referral burden falls evenly across the screened population, and Section 5.5 shows that it does not. At the operating point examined there, false positives run at 2.5% among participants for whom Spanish is not the native language and 30.1% among native speakers. A referral total is therefore an average over two groups experiencing very different burdens, and the aggregate figure conceals rather than characterises the instrument’s behaviour. We give the aggregate for comparability with the original submission, but the per-stratum rates are the ones that bear on deployment: an instrument that unnecessarily assesses three in ten unaffected children in one language group and one in forty in another should not be deployed on the strength of its pooled referral count.

The original manuscript argued that repeated annual screening would compound the detection probability, reducing a 55% miss rate to $0.55^{3}=16.6\%$. We withdraw this argument: it assumes independence across screenings of the same child on the same instrument, whereas misses are strongly correlated within-child. A child whose performance profile the model reads as typical will tend to read as typical again. Additional referrals based on teachers’ comments, questionnaires to parents, or quick phonological awareness tasks may lead to the identification of at-risk children who have been missed by the automated screen. The Brier score improvement (0.0690 vs. 0.0818, ie, 15.6% reduction in calibration error) is of clinical importance since better-calibrated probability estimates provide for risk-stratified referral decisions: instead of using a fixed binary threshold, clinicians would be able to refer all children who are above a high-confidence threshold right away and at the same time keep monitoring those in the intermediate-risk group by using follow-up assessments.

Besides binary classification, a clinical use of graph-theoretic features is that they can be seen as interpretable markers of an individual’s cognitive profile: for example, if a child’s performance difference between high-core and low-core tasks (core-weighted) is very large, this can lead to the suspicion that the child’s central cognitive integration is specifically affected compared to peripheral processing, which can help indicate the choice of intervention methods which focus on integration skills instead of the individual component skills.

### 6.4 Limitations and threats to validity

Several limitations of this study need to be discussed explicitly. First, in terms of external validity, this framework has only been tested on one dataset (Dytective) comprising Spanish-speaking participants given one gamified task battery. This constrains our negative result as much as it would have constrained a positive one: what we have shown is that graph-derived features add nothing detectable *for this battery in a transparent orthography*, and a null of that kind does not generalise automatically. Spanish maps graphemes to phonemes with high consistency, so phonological deficits surface differently than in opaque orthographies such as English, where decoding difficulty is more visible in accuracy and less in speed. A task-correlation network estimated in an opaque orthography could plausibly carry structure that ours does not. Dyslexia shows different patterns in orthographies with different degrees of transparency - the difficulties of readers of more transparent orthographies such as Spanish are quite different from those in less transparent ones such as English or Chinese. It is still an open question whether the network structural patterns identified in this study can be generalized to other languages, different task batteries, and cultural contexts. This issue can only be resolved through validation using multiple datasets.

Second, the 8.3:1 class imbalance is consistent with population prevalence but limits attainable sensitivity. Both arms detect roughly half of affected participants (51.3% and 56.4%), which is low for any diagnostic purpose and marginal even for first-stage screening. Class-weighted classifiers mitigate this only partially; the underlying difficulty is detecting a minority class under strong skew, and it constrains both arms equally.

Third, the topological analysis part is based on a very basic approach Betti numbers and filtration curves as opposed to the full persistent homology with persistence diagrams, persistence landscapes, or persistence images. Full persistent homology—essentially finding birth-death pairs for topological features throughout the filtration—can encapsulate a richer layer of structural information, including feature lifetimes and their scatter. GUDHI, Ripser, or giotto-tda are some of the libraries that could facilitate this extension and might lead to discovering more discriminative topological features.

Fourth, the correlation threshold τ=0.25 was adopted without systematic optimisation, and the threshold determines the network topology: lower values give denser, more clustered networks, higher values retain only the strongest correlations. Our original submission listed a sensitivity analysis as desirable future work; we have since performed it (Section 5.1, Table 3), and it is the reason the population-level structural claims are withdrawn. Five of six group differences reverse sign across τ∈[0.15,0.45]. What remains unaddressed is the choice of τ itself: we report results across a range rather than defending a single value, and a principled criterion for selecting one would require external validation the present data cannot supply.

Fifth, and most seriously, the protocol reported in the original submission selected its operating point by minimising the McNemar p-value over 1,320 configurations evaluated against the test labels, and selected its comparator so as to maximise the errors the ensemble could correct. Neither quantity was a valid statistic. This has been corrected rather than mitigated: selection now occurs on a separate validation partition, the comparator is chosen by cross-validated AUC on training data alone, and the test partition is read once. We note for completeness that the resulting analysis reverses the paper’s original conclusion.

Sixth, three defects in the analysis pipeline are known to us and are disclosed here rather than left for a reader to find. (i) The GRAPHOS operating point selected on validation, τ=0.05, lay at the lower edge of the threshold grid searched; the unconstrained optimum may therefore lie below the grid, meaning that operating point was partly set by the grid boundary rather than by the data. The same applies to two of the comparator arms in Table 6. Because our primary endpoint is threshold-free this does not affect the headline result, but it does affect every threshold-dependent quantity we report. (ii) The ensemble was assembled from the ten highest cross-validated-AUC models *excluding* the comparator, so the single strongest model was unavailable to it. For a null result this is conservative—the ensemble was, if anything, handicapped—but a reviewer is entitled to note that we did not give the proposed method its strongest form. (iii) Eight volatility features were discarded as zero-variance owing to a missing-value artifact in a rolling-window computation rather than genuine absence of variation; the feature space we report as 282 columns would be 290 absent that defect. A corrected implementation addressing all three is available with the code, and we would rerun on request; we do not expect the conclusions to change, since the AUC difference across ten splits is +0.0042 with a confidence interval spanning zero.

Seventh, and in our view most consequentially for anyone using this dataset, performance is substantially attributable to a demographic confound rather than to dyslexia-related task performance. Section 5.5 shows that dyslexia prevalence in the sample differs 4.7-fold by native-language status (5.38% vs. 25.38%, $p=2.2\times 10^{-40}$), that four demographic columns alone reach AUC 0.7241 against 0.7443 for all 192 task metrics, and that including them produces a twelve-fold disparity in false-positive rate between language groups (2.5% vs. 30.1%). We had not examined this in the original submission, and it changes how the reported accuracies should be read.

The consequence for deployment is direct and we state it plainly: an instrument with these properties would refer roughly three in ten unaffected native-speaking children while referring one in forty unaffected non-native-speaking children. That is not a defensible screening characteristic, and it is a fairness problem independent of any question about graph features. Removing the native-language column halves the disparity but does not eliminate it, because task performance itself covaries with language background; stratified modelling or explicit fairness constraints would be required, and we have not attempted either here.

We also note the implication for the wider literature without overstating it. Published classification accuracies obtained on this dataset using demographic covariates alongside task metrics may reflect this prevalence imbalance to an extent that has not, so far as we are aware, been quantified. We do not claim that any specific prior result is invalid; we report that the confound is present, that it is large, and that establishing how much of any reported accuracy survives its removal requires the stratified analysis we set out above.

Eighth, two residues of Reviewer 2’s concerns remain open. (a) The published documentation for the battery provides no item-level mapping of subtests to processing constructs, and our own factor analysis supplies no empirical substitute: 28 of 32 tasks load on no factor, so the data do not license a construct grouping either. A properly grounded neuropsychological account would require an independently established mapping. (b) Test–retest reliability cannot be estimated, because the dataset contains no repeated administrations. We have estimated the remaining psychometric quantities directly (Section 4.2).

Ninth, all results derive from a single dataset. A negative result constrains less than a positive one would, and the demographic confound in particular is a property of this sample’s recruitment rather than a general claim about the battery; both require independent data to establish their generality. We note also that the ablation of Table 9 uses the corrected 290-column feature construction while the headline comparison uses the 282-column version reported in Section 4.4; the two are consistent in direction, but a fully unified rerun has not been performed.

**Table 9:** Leave-one-group-out ablation, scored on the validation partition with the model class held fixed. Positive Δ means removing the group degrades discrimination. p-values are Holm-adjusted across the family.

Removed	Features	Val AUC	ΔAUC	p (Holm)
– (full model)	290	0.8389	–	–
Demographics	286	0.7295	+0.1094	**0.0001**
Graph signal processing	278	0.8327	+0.0062	1.000
Hierarchical decomposition	283	0.8368	+0.0021	1.000
Pattern entropy	288	0.8370	+0.0019	1.000
Cross-metric consistency	285	0.8377	+0.0013	1.000
All graph-derived (GSP + HGD)	271	0.8438	−0.0049	1.000
Profile statistics	226	0.8448	−0.0058	1.000
Sequential structure	286	0.8458	−0.0069	1.000
All 192 raw task metrics	98	0.8598	−0.0209	1.000

Bold values indicate the superior result in each metric.

Finally, the computational pipeline, despite being executable on standard hardware (runs in a few minutes on a single NVIDIA RTX A4000 GPU), is quite elaborate: six analytical components, 282 features of which only 19 are graph-derived, 29 candidate classifiers, and multi-strategy aggregation. Such a level of complexity can make it quite difficult to reproduce, certify in a clinical setting and deploy in environments with limited resources. However, a less complicated variant of the framework that only keeps the most discriminatory graph-theoretic elements might be a way of getting around this problem.

## 7 Conclusion

This article introduced GRAPHOS, a graph-theoretic framework for dyslexia detection that builds task correlation networks from gamified cognitive assessment data and retrieves multi-scale structural features via six population-level analytical modules: Network Motif Discovery, Topological Feature Analysis, Graph Signal Processing, Hierarchical Graph Decomposition, Network Robustness Analysis, and Multi-Resolution Graph Analysis.

Individual-level features are obtained by network alignment. Contrary to our original report, the resulting ensemble does not improve on a strong single-classifier comparator: the primary threshold-free comparison returns ΔAUC=−0.0001 (p=0.989), and the null replicates across ten random splits. Nineteen of 282 features are graph-derived, and removing them does not significantly degrade discrimination.

The hypothesis motivating the framework—that task inter-correlation structure holds diagnostic information which flat feature representations discard—is therefore not supported by these data. Combining several graph-theoretic paradigms does yield a larger feature space, but a larger feature space is not the same as a more informative one, and on the evidence here it was not.

Our original submission reported population-level differences between the dyslexic and non-dyslexic task networks—reduced transitivity, disassortative mixing, elevated spectral entropy, reduced modularity, elevated global efficiency—and interpreted them as evidence of disrupted cognitive integration. Against a size-matched null constructed from 1,000 subsamples of the larger group, none of nine such metrics survives, and across the correlation-threshold range five of six differences reverse sign. These findings are withdrawn. What remains is the control itself: comparing correlation networks between groups of unequal size at a fixed threshold confounds topology with sampling precision in a predictable direction, and we recommend the subsampling procedure of Section 5.1 as a routine safeguard. A third finding emerged from examining which features carry the model’s discrimination. Dyslexia prevalence in this dataset differs 4.7-fold by native-language status, four demographic columns alone reach AUC 0.7241 against 0.7443 for all 192 task metrics, and including them produces a twelve-fold disparity in false-positive rate between language groups. We regard this as the most consequential result of the revision: it bears on any classifier trained on this dataset, not only on ours, and it means that reported accuracies which pool across language strata require a stratified re-examination before they can be read as measures of dyslexia detection.

The lists of future work can be testing GRAPHOS on multilingual datasets and different task batteries; using specialized libraries for full persistent homology computation; perform longitudinal tracking of network evolution during intervention; investigating graph neural network learning directly from task networks; exploring individual graph-theoretic features as interpretable biomarkers that can guide personalized intervention; and a principled criterion for selecting the correlation threshold, since the sensitivity analysis reported here establishes that the choice matters but not how it should be made.

## Code availability

The full analysis pipeline, including the corrected implementation and the run manifest recording seeds, package versions, and every selection decision, is available from the corresponding author on request.

## Data Availability

The original contributions are included in the article, and further inquiries can be directed to the corresponding author.
